# Strategies to support university employee and student caregivers of older adults

**DOI:** 10.1093/geroni/igaf122.2356

**Published:** 2025-12-31

**Authors:** Jodi Southerland, Erin Mauck, Shimin Zheng, Esther Osime, Kwangman Ko, Matthew Smith

**Affiliations:** East Tennessee State University, Johnson City, Tennessee, United States; East Tennessee State University, Johnson City, Tennessee, United States; East Tennessee State University, Johnson City, Tennessee, United States; East Tennessee State University, Johnson City, Tennessee, United States; East Tennessee State University, Johnson City, Tennessee, United States; Texas A&M University, College Station, Texas, United States

## Abstract

A growing number of university employees and students provide care to older adults. These individuals often face unique challenges navigating their professional, academic, and caregiving responsibilities. While universities can adapt their culture to meet caregivers’ diverse needs, limited research identifies caregivers’ perspectives about university-based support strategies necessary to promote caregiver-friendly environments. The study objective was to explore strategies for supporting caregivers in a university setting using a mixed methods approach. A Qualtrics survey was administered to 143 employees and 123 students who provide care to an individual >55 years at an Appalachian university. Questions examined demographic characteristics, caregiving context, and support strategies. Survey responses were analyzed for descriptive statistics. Individuals who completed the survey were invited to participate in semi-structured interviews to gain further insight into their responses. Sixteen individuals (9 employees and 7 students) participated in interviews. Thematic analysis was used to identify key support strategies. Flexibility (63.9%), general resources for caregivers (39.1%), information about university resources (37.6%), self-care (27.8%), and counseling (19.9%) were the most cited assistance that universities could provide to help caregivers. Employees were more likely to report needing information about general and university-specific resources. Qualitative analysis supported these findings and revealed new concepts such as a need to promote awareness about caregivers as a “hidden” group. Findings highlight the needs of employee and student caregivers and identify actionable support strategies to enhance caregiver well-being on campus. Strategic planning and administrative support are needed to implement such strategies and create caregiver-friendly environments in university settings.

